# Thermal Conductivity Performance of 2D h-BN/MoS_2_/-Hybrid Nanostructures Used on Natural and Synthetic Esters

**DOI:** 10.3390/nano10061160

**Published:** 2020-06-12

**Authors:** Jaime Taha-Tijerina, Hélio Ribeiro, Karla Aviña, Juan Manuel Martínez, Anna Paula Godoy, Josué Marciano de Oliveira Cremonezzi, Milene Adriane Luciano, Marcos Antônio Gimenes Benega, Ricardo Jorge Espanhol Andrade, Guilhermino José Macedo Fechine, Ganguli Babu, Samuel Castro

**Affiliations:** 1 Departamento de Ingeniería, Universidad de Monterrey, Av. Ignacio Morones Prieto 4500 Pte., San Pedro Garza García, NL 66238, Mexico; karla.avina@udem.edu (K.A.); juan.martineza@udem.edu (J.M.M.); 2 Mackenzie Institute for Research in Graphene and Nanotechnologies-MackGraphe, Universidade Presbiteriana Mackenzie, Rua da Consolacão, 896, Sao Paulo, SP CEP 01302-907, Brazil; helio.ribeiro1@mackenzie.br (H.R.); annapsgodoy@hotmail.com (A.P.G.); josuecremonezzi@hotmail.com (J.M.d.O.C.); 71965629@mackenzista.com.br (M.A.G.B.); ricardo.andrade@mackenzie.br (R.J.E.A.); guilherminojmf@mackenzie.br (G.J.M.F.); 3 Departamento de Química, Universidade Federal de Minas Gerais, Av. Antonio Carlos, 6627, Pampulha, Belo Horizonte, MG CEP 31270-901, Brazil; mileneluciano@yahoo.com.br; 4 Materials Science and NanoEngineering, Rice University, 6100 Main St., Houston, TX 77005, USA; (babu.ganguli@rice.edu (G.B.); samuel.castro@rice.edu (S.C.)

**Keywords:** thermal conductivity, 2D structures, boron nitride, molybdenum disulfide, hybrid, nanolubricants, esters

## Abstract

In this paper, the thermal conductivity behavior of synthetic and natural esters reinforced with 2D nanostructures—single hexagonal boron nitride (h-BN), single molybdenum disulfide (MoS_2_), and hybrid h-BN/MOS_2_—were studied and compared to each other. As a basis for the synthesis of nanofluids, three biodegradable insulating lubricants were used: FR3^TM^ and VG-100 were used as natural esters and MIDEL 7131 as a synthetic ester. Two-dimensional nanosheets of h-BN, MoS_2,_ and their hybrid nanofillers (50/50 ratio percent) were incorporated into matrix lubricants without surfactants or additives. Nanofluids were prepared at 0.01, 0.05, 0.10, 0.15, and 0.25 weight percent of filler fraction. The experimental results revealed improvements in thermal conductivity in the range of 20–32% at 323 K with the addition of 2D nanostructures, and a synergistic behavior was observed for the hybrid h-BN/MoS_2_ nanostructures.

## 1. Introduction

For over a century, fossil fuels have been the major contributor to the energy sector. Challenges to mitigate pollution, global warming, and more will be critical. This, together with the imminent scarcity of oil reserves, increases in oil prices, rises in lubricant disposal costs, as examples, will promote the development of alternative sources of energy as well as novel technologies to attend the current and future world energy needs with sustainability and efficiency [1,2,3].

Petroleum-based fluids and lubricants are applied as lubricants and dielectric and coolant material in electrical or electronic devices such as power systems (transformers), machinery, and the automotive and biomedical industries, among others [4,5,6,7]. Paraffinic and naphthenic oils are examples of mediums used for these applications [8]. Nevertheless, these materials exacerbate environmental issues due to their eco-toxicity, incomplete biodegradability, and highly probable carcinogenic characteristics [9,10]. Some of the main limitations to be solved regarding these applied fluids are how to dispose of them and prevent products to have a significant negative impact on health and environmental-related consequences, i.e., skin and respiratory diseases or in case of a spillage [11,12,13,14].

More often, biodegradable synthetic products are used in environmentally-sensitive areas such as electric, electronic, automotive, agricultural, and biomedical industries [4,5,6,7,15,16,17]. The increased environmental awareness is a primary driving force for the novel technological developments in these fields [11,18,19]. Synthetic and natural esters are an alternate to petroleum-based oils because they have non-toxic attributes and are renewable and environmentally-friendly. Moreover, synthetic esters are highly resistant to oxidation [20,21] and have high fire points. It has been proven that these fluids reduce hydrocarbon and carbon monoxide emission levels, which is the reason why their use has been increased recently in the industry. Furthermore, they possess great characteristics such as excellent lubricity, high thermal conductivity, high flash and fire points, and biodegradability, as compared to petroleum-based fluids. Nevertheless, despite specific environmental benefits of natural or vegetable fluids, their characteristics do not always accomplish the required properties [22].

Due to their inherent risks, such as fire and pollution (caused by leakage and spills), natural alternatives derived from triglycerides in vegetable oils and animal fat are being sought [23,24,25,26]. Although a great sustainable feedstock for novel materials, these are susceptible to variations in their compositions due to different processing, crop conditions, and climate and environment, among others [27]. In general, the natural esters are presented as fatty acids, containing the ester function and a long aliphatic chain that dictate some chemical and mechanical properties of the oils [28]. The oils based on saturated fatty acids are more chemically stable and very viscous. Unsaturated (mono, di, tri) can be much less viscous but are prone to oxidation [28], which leads to an increase in viscosity and consequently affect thermal, tribological, and other properties of the oil [29].

In industrial and thermal systems, heat dissipation is a paramount phenomenon. Devices, machines, and equipment miniaturization design require better performance (thermal dissipation efficiency) of fluids and lubricants due to environmental challenges and global market competition [22,30]. Several heat transfer fluids have been under research with the aid of nanoreinforcements to improve their performance [4,5,31,32,33,34]. In recent years, environmental awareness has gained increased attention and is a driving force towards the novel technology and innovation developments in diverse fields. Biodegradability has become a key design factor and request for products. In this sense, a good candidate to replace petroleum-based oils are synthetic or natural esters based on vegetable oils such as sunflower, coconut, corn, soybean, rapeseed, olive, palm oil, and others [18,19,30,35,36]. These materials are an alternative to mineral oils because they have non-toxic properties and are renewable and environmentally-friendly.

Two-dimensional nanomaterials appear as an attractive alternative due to the high surface area they possess for heat transfer. Among these 2D nanomaterials are hexagonal boron nitride (h-BN) and molybdenum disulfide (MoS_2_), which are suitable for development of high-performance composites applied in several industrial and technological sectors due to their exceptional electrical, thermal, and mechanical properties [37,38,39]. In this sense, hexagonal boron nitride (h-BN) is investigated in this study, since it is applied to several substrates to reduce viscosity and increase thermal conductivity [7,24,37,38,39,40,41]. The capability of h-BN to improve the thermal conductivity of solid nanocomposites based on epoxy and polyurethane was previously reported by Ribeiro et al. [37,38,39]. Nonetheless, the application of h-BN to mineral oils, natural esters, and other fluids, is being studied [5,7,26,42]. Taha-Tijerina et al. [7] showed that h-BN was able to improve the thermal conductivity of a mineral oil, and—differently to graphene—functioned as an electric insulator rather than a conductor. Such phenomena is due to the ability of some nanoparticles, such as F_3_O_4_ [25], h-BN [25], Al_2_O_3_ [43], SPION [44], CuO [43], and BaTiO_3_ [45] to trap moving electrons. Du et al. [42] applied boron nitride (BN) to a vegetable oil and found that the thermal conductivity was increased in comparison with the pure oil. The use of BN allowed the lubricant to be less viscous at higher temperatures which further enhanced the temperature dissipation. Salehirad and Nikje [5] reported similar results applying h-BN to a mineral oil: increased thermal conductivity, lower viscosity, and better insulation capability when compared to the pure mineral oil. Li et al. [46] developed ethylene glycol/boron nitride nanofluids, varying the size of the nanoparticles. It was demonstrated that nanofluids containing larger nanoparticles have higher improvements in thermal conductivity than smaller nanoparticles. Ilhan et al. [47] reported an enhancement of thermal conductivity for h-BN nanofluids of up to 26%, 22% and 16% at 3 vol.% for water, water/ethylene glycol mixture, and ethylene-glycol-based nanofluids, respectively.

Molybdenum disulfide (MoS_2_) involves very optimal thermal and chemical stabilities. It demonstrates applications as a catalyst and lubricant due to its unique properties like anisotropy, photocorrosion resistance and chemical inertness [48,49]. MoS_2_ nanostructures have good thermo-physical features and great anti-friction properties, making it another promising material as reinforcement for the cooling purposes of nanofluids. Diverse factors such as the composition and loading of nanoparticles, suspension stability, base fluid composition, nanofluids preparation method, surface modifier, and surfactant application may influence the thermo-physical properties of nanofluids, including viscosity and thermal transport [50,51,52,53,54,55]. Su et al. [52] investigated stability and thermal transport behavior of MoS_2,_ water-based, and oil-based nanofluids at various concentrations (0.01 wt.% to 0.5 wt.%). It was observed that by increasing the filler fraction, the thermal conductivity increased. Furthermore, the thermal conductivity improvement in MoS_2_ and water-based nanofluids was higher than for oil-based nanofluids, which according to Su et al. was due to a better dispersion and suspension stability achieved on water-based fluid. Zeng et al. found that thermal conductivity of dibenzyl-toluene-based MoS_2_ nanofluids increased from 17.5 to 37.5% as the temperature increased from 40 to 180 °C [53].

In this work, the effect of incorporation and homogeneous dispersion of single h-BN, MoS_2_, and hybrid h-BN/MoS_2_ mixture at various concentrations (by weight) within synthetic and natural esters, aims to improve their thermal conductivity performance, which is determined at various evaluating temperatures (up to 323 K). The contributions of this work rely on the aspect of biodegradable materials suitable for industrial applications, such as energy and metal-mechanic field. The nanofluids with only h-BN or MoS_2_ nanofiller components were compared with their hybrid h-BN/MoS_2_ combination.

## 2. Materials and Methods

In our study, synthetic esters Midel 7131 (M&I Materials-Manchester, UK) and natural esters—Envirotemp^®^ FR3™ (Cargill Industrial Specialties–Minneapolis, MN, USA) and VG-100 (Prolec GE International, Apodaca, México) [56] (Table 1)—were used as base materials to develop different nanofluids with h-BN, MoS_2_, and h-BN/MoS_2_ nanostructures (Table 2) at various concentrations of 0.01, 0.05, 0.10, 0.15, and 0.25 wt.%. The nanofluids with the hybrid composition h-BN/MoS_2_ were used at a mass ratio of 1:1 between h-BN and MoS_2_. Nanofillers were obtained by wet exfoliation in tetrahydrofuran (THF), using probe ultrasound for 5 h at an amplitude of 35%. The exfoliated material was filtered and dried in a vacuum oven for 24 h at 80 °C. To prepare the nanofluids, a two-step method to homogeneously disperse the 2D nanostructures within the esters was used. Glass containers were prepared with each set of fluids at different filler fractions: 0.01, 0.05, 0.10, 0.15, and 0.25 wt.% of the nanostructures for each set of fluid. Extended water bath sonication (~4–5 h) was used (Branson ultrasonic homogenizer model 5510-Danbury, CT, USA, 40 kHz). To avoid the nanostructures’ agglomeration and quick sedimentation, the temperature of the water bath was maintained constant at room temperature (24 °C). Samples were maintained on a drawer for at least 2 weeks without significant sedimentation. Experimental evaluations were performed after 3 days of sample preparation. This process assists in obtaining the stable homogeneous nanofluids, which are further evaluated.

Nanomaterials’ morphology and thickness were analyzed by atomic force microscopy (AFM) using an Asylum Research MFP-3D-AS (Belo Horizonte, MG, Brazil), operated in contact mode. An Olympus AC240TS probe (Belo Horizonte, MG, Brazil) and a 70 kHz resonant frequency were used. Specimens were prepared by ultrasonically dispersing the 2D nanostructures in isopropanol (IPA) and water mixture. This was followed by the deposition of dispersion drops on mica, which was then dried. A Quanta™ 200 FEG-FEI microscope (Belo Horizonte, MG, Brazil), operated under a vacuum with a 30.0 kV accelerating voltage, was used to obtain SEM images. The 2D nanostructures were prepared by dispersing the material in IPA/water mixture using an ultrasonic water bath (2 h); subsequently, the sample was cast onto a silicon substrate and evaporated in air at room temperature. The chemical composition of the 2D nanostructures was investigated (EDS) using a FEI QUANTA™ 200 microscope (Belo Horizonte, MG, Brazil). To evaluate the homogeneity of the mixture between them, EDS mapping was performed.

**Figure 1:**
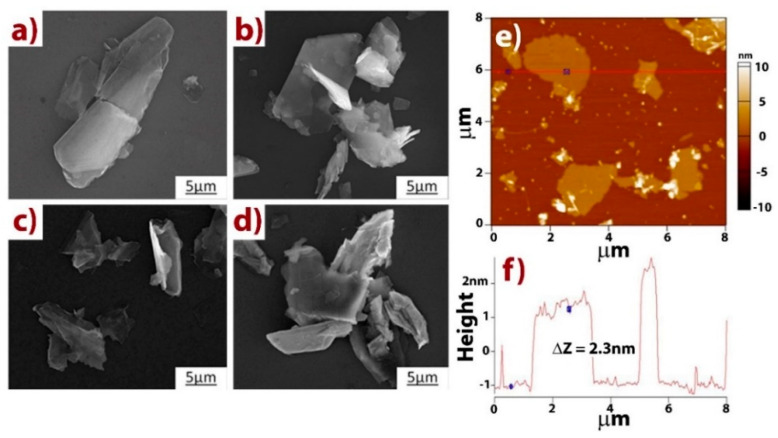
SEM images of h-BN (**a**), MoS_2_ (**b**), and h-BN/MoS_2_ nanosheets (**c**,**d**). AFM image of the h-BN/MoS_2_ mixture and its height profile (**e**,**f**).

SEM was also performed to characterize the microstructure of the h-BN/MoS_2_ mixture. Figure 1a–d shows SEM images of h-BN, MoS_2_, and h-BN/MoS_2_ nanosheets with different morphologies and sizes. Figure 1a shows a typical SEM image of a large-size h-BN sample, which consists of thin and crumpled sheets with a lateral dimension of about ~5–20 µm. Figure 1b shows the morphology of MoS_2_. Figure 1c,d shows a typical SEM image of the h-BN/MoS_2_ mixture, which keeps its features between its single components. Finally, Figure 1e–f presents the AFM image of h-BN/MoS_2_ and its corresponding height profile (thickness approximately 2.3 nm).

**Figure 2:**
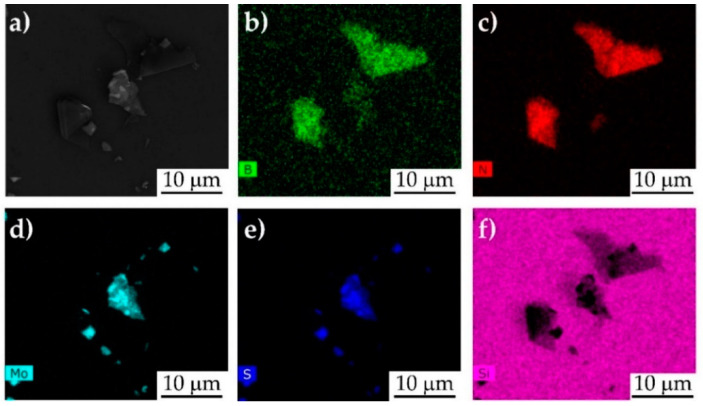
SEM image of h-BN/MoS_2_ system (**a**) and its resulting EDS mapping of the elements B, N, Mo, and S on SiO_2_ grid (**b**–**f**).

The h-BN/MoS_2_ mixture can be also confirmed by EDS maps obtained from the SEM image (Figure 2a), which displays the homogeneity of the mixture of h-BN and MoS_2_. Elements B, N, Mo, and S are uniformly distributed in the whole area according to Figure 2b–e.

**Figure 3:**
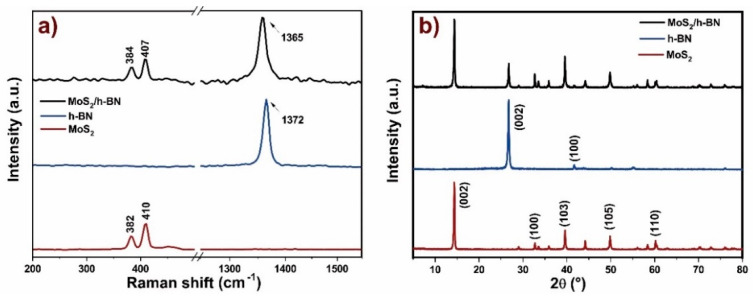
(**a**) Raman and XRD spectra of h-BN, (**b**) MoS_2_ and the h-BN/MoS_2_ mixture.

Raman spectra of the h-BN nanostructures were obtained by a Raman Confocal Spectrometer WITec, model Alpha 300R (Sao Paulo, SP, Brazil), using a laser (532 nm) as the excitation source. The MoS_2_ nanosheets on the Raman spectrum showed the main characteristic bands of this nanostructure (Figure 3a). The first one, in 382 cm^−1^, is attributed to E_2g_ vibrational mode, and the second (in 410 cm^−1^) is related to A_1g_ mode. On the other hand, these modes correspond to the in-plane vibrations of sulfur atoms in one direction and molybdenum atoms in another one, and to out-of-plane vibrations (A_1g_) of sulfur atoms. For the sample containing h-BN, a spectrum with only one intense band in 1372 cm^−1^, in relation to the E_2g_ vibrational mode, was observed. In relation to the mixture of these nanofillers, the same three bands from MoS_2_ and h-BN nanosheets were presented, without another additional band or significative shifting from original positions related from the original nanomaterials. [37,63].

X-ray diffraction (XRD) evaluation was performed using a Shimadzu XRD-7000 system (Sao Paulo, SP, Brazil) employing Cu Kα radiation (λ = 1.5418 Å). The XRD patterns were obtained in the 2*θ* range between 4° and 80° at a scanning rate of 4° min^−1^. These results were found to be fully matched with a JCPDS 37–1492 card. The XRD spectrum of MoS_2_ presented diffraction peaks in *2θ* equal to 14.4°, 32.8°, 39.6°, 49.8°, and 60.3° related to (002), (100), (103), (105), and (110) planes, respectively. The diffractogram of h-BN depicted peaks in *2θ* equal to 26.7° and 41.7° from the (002) and (100) planes (Figure 3b) [38]. The mixture presented the same peaks related to both nanofillers, without additional peaks or significative shifting from the original MoS_2_ or h-BN diffraction spectra.

Thermal conductivity measurements were carried out on nanofluids at various nanostructure concentrations according to the transient hot-wire (THW) technique, with a KD2 Pro device. A temperature-dependence scan was also performed, maintaining a thermal equilibrium for at least 10 min before each set of evaluations. A minimum of 7 measurements were taken for each set of experiments to report average values with standard deviation as error bars.

## 3. Results

**Figure 4:**
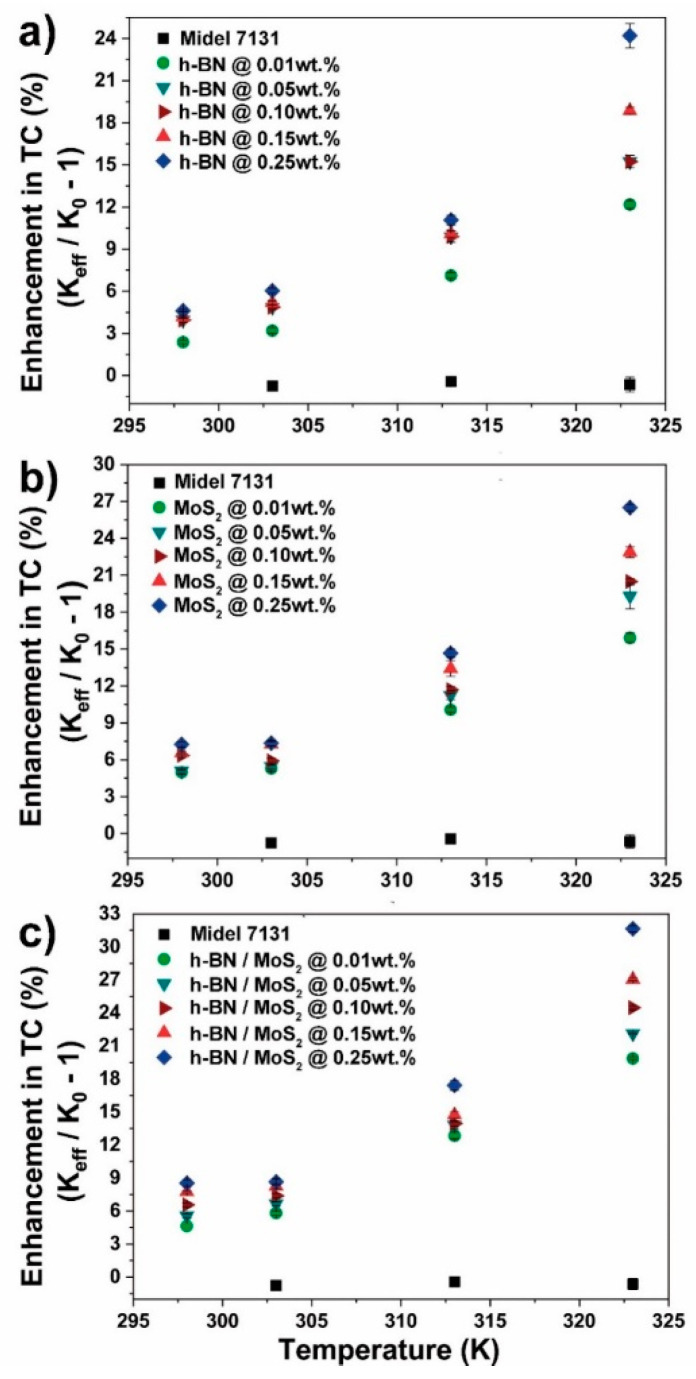
Conductivity performance of Midel 7131: (**a**) h-BN, (**b**) MoS_2_, and (**c**) h-BN/MoS_2_ nanofluids under temperature-dependence evaluation (percentage of filler amount is mentioned).

Improvements in thermal conductivity in synthetic and natural ester systems have been obtained for different types of nanofillers. Figure 4 depicts the temperature-dependent thermal conductivity performance of Midel 7131 nanofluids with h-BN, MoS_2_, and h-BN/MoS_2_ at various filler fractions. The synthetic ester did not show significant temperature dependency (less than 1% at 50 °C): actually, a decrement in thermal conductivity was observed as temperature was increased, similar to other authors’ findings [64,65]. In general, the thermal conductivity of the evaluated nanofluids gradually increased for all the fillers and concentrations studied as temperature was also increased.

Figure 4a depicts the effect of h-BN on the thermal conductivity of Midel 7131. As filler fraction and temperature is increased, thermal conductivity also increases. For instance, at 323 K, improvements of 12, 14, 15 and 19% are obtained at 0.01, 0.05, 0.10, and 0.15 wt.%, respectively. A maximum enhancement of 24.2% in thermal conductivity was observed at 0.25 wt.% at 323 K. A similar improvement trend was observed for the nanofluids with MoS_2_ addition (Figure 4b), showing higher enhancement than h-BN reinforcement. Here, the improvement was 16, 19, 20.5, 23 and 26.5% at 0.01, 0.05, 0.10, 0.15, and 0.25 wt.%, respectively. Thermal conductivity showed significant improvement when hybrid nanostructures were applied as reinforcement. In this case, the incorporation of h-BN/MoS_2_ nanofiller, contributed to improvement, was 20, 22, 24.5, 27 and 32% at 0.01, 0.05, 0.10, 0.15, and 0.25 wt.%, respectively (Figure 4c).

**Figure 5:**
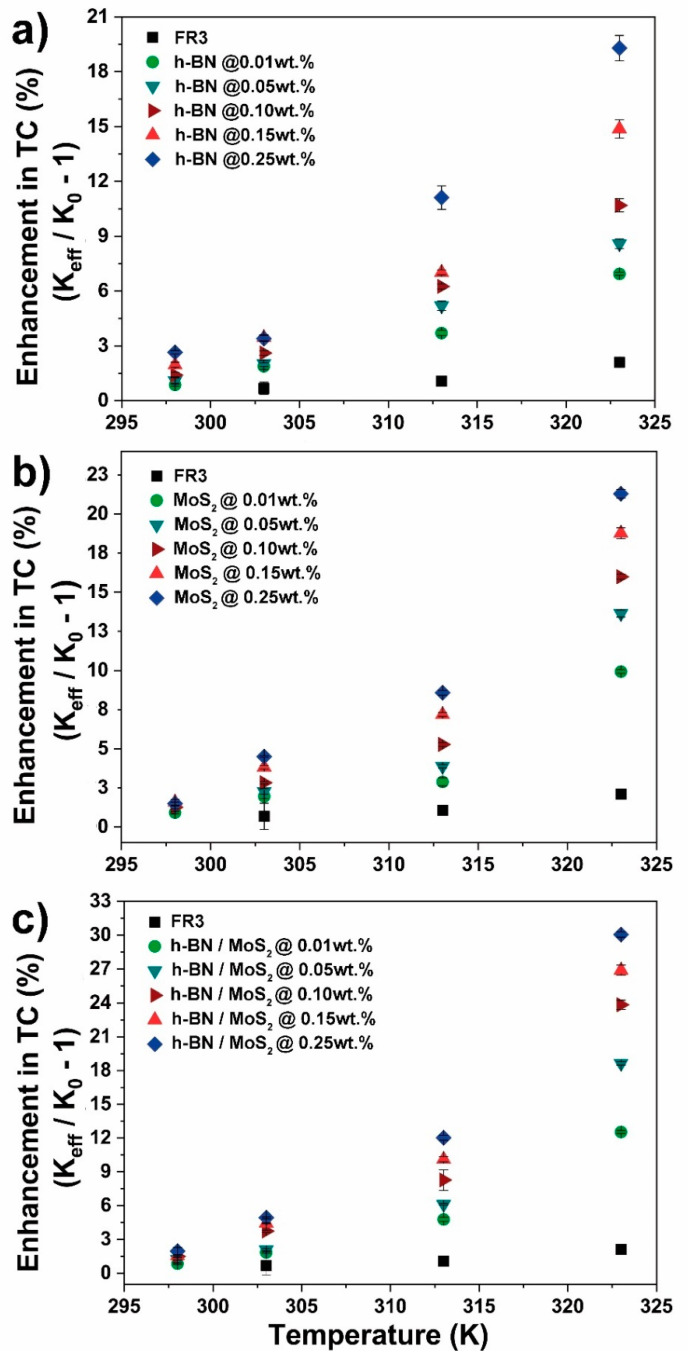
Conductivity performance of FR3: (**a**) h-BN, (**b**) MoS_2_, and (**c**) h-BN/MoS_2_ nanofluids under temperature-dependence evaluation (percentage of filler amount is mentioned).

**Figure 6:**
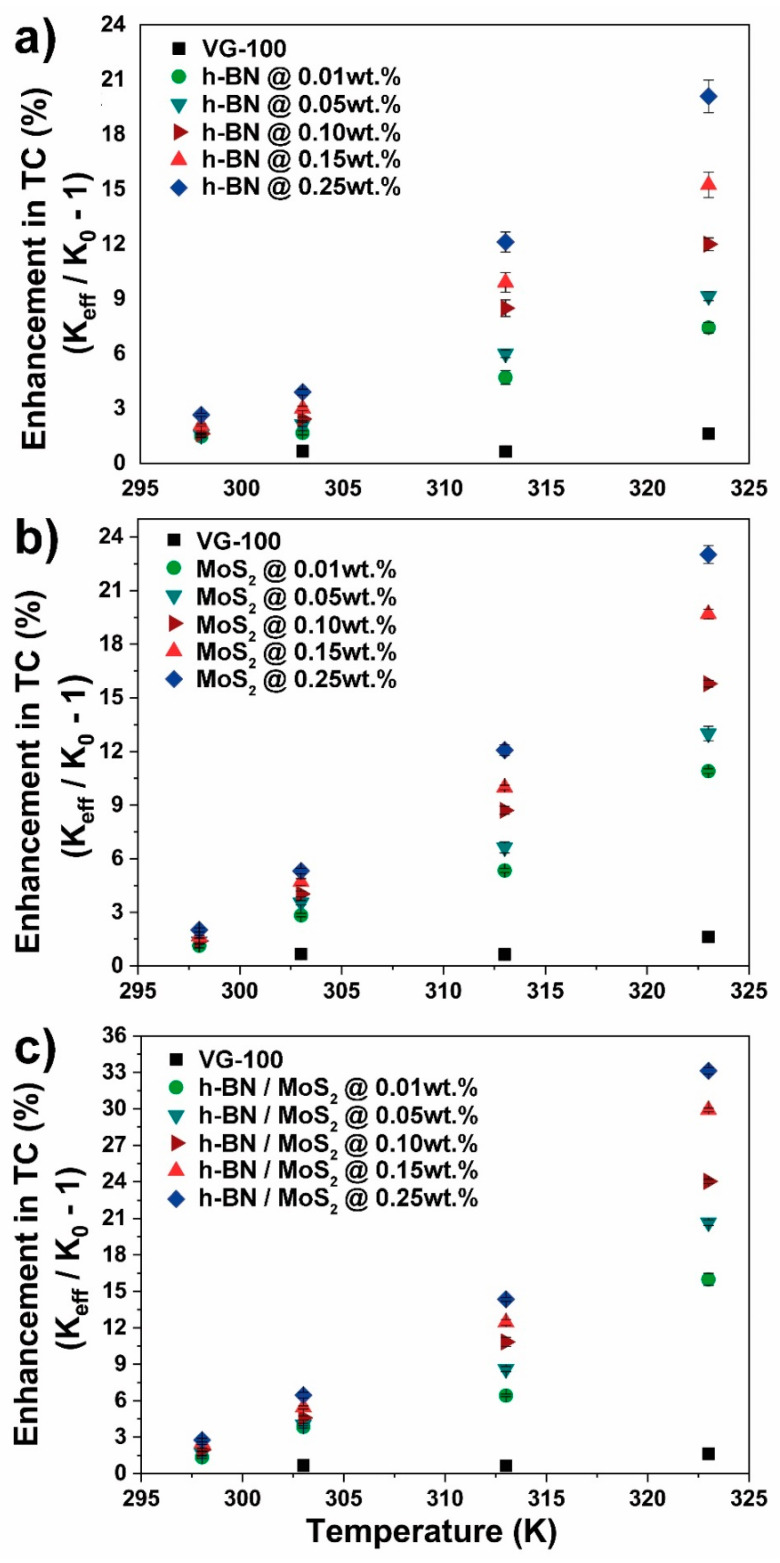
Conductivity performance of VG-100: (**a**) h-BN, (**b**) MoS_2_, and (**c**) h-BN/MoS_2_ nanofluids under temperature-dependence evaluation (percentage of filler amount is mentioned).

Natural ester systems are shown in Figure 5 (FR3) and Figure 6 (VG-100). These two green fluids displayed very similar levels of improvement for all fillers. Figure 5a and Figure 6a show the effect of h-BN-filler fraction and temperature rise, reaching a maximum improvement of ~20% in thermal conductivity at 0.25 wt.% at 323 K.

The addition of MoS_2_ within natural esters showed an improvement trend similar to h-BN (Figure 5b and Figure 6b). In this case, thermal conductivity enhancement of 10, 14, 16, 19 and 21% at 0.01, 0.05, 0.10, 0.15, and 0.25 wt.% was observed for FR3 fluid (Figure 5b). Moreover, VG-100 nanofluids showed a slightly higher enhancement, reaching a maximum of 23% at 0.25 wt.% at 323 K (Figure 6b).

The incorporation of the h-BN/MoS_2_ nanofiller to natural esters resulted in significant improvements in thermal conductivity. Achieving enhancements of 13, 19, 24, 27 and 30% at 0.01, 0.05, 0.10, 0.15, and 0.25 wt.% was observed for FR3 fluid (Figure 5c). Moreover, enhancements of 16, 21, 24, 30 and 32% at 0.01, 0.05, 0.10, 0.15, and 0.25 wt.%, respectively (Figure 6c), were observed for VG-100 nanofluids.

The incorporation of 2D nanostructures significantly improves the performance of the nanofluids. h-BN contributes to enhance thermal conductivity and MoS_2_ also contributes to the fluid’s reinforcement and may help to obtain an homogenous dispersion of the nanofillers due to its well-known lubricant properties [66,67]. It is suggested that due to the low filler fraction used, the observed enhancement on thermal conductivity is due to molecular interactions (collisions) between the fluid and the 2D nanostructures [7,14,37,53,68,69]. However, observing thermal conductivity behavior during the temperature-dependent evaluations indicates that this increase is based on the percolation mechanism as well as the contribution of the Brownian motion of the sheet-like nanostructures [70,71,72]. As the concentration of 2D nanostructures is increased in the synthetic or natural esters, the chance of phonons to get scattered in the contiguous nanostructures increases proportionally, which leads to enhanced contact conductance [73]. Consequently, thermal conduction channels are formed, which may increase the thermal conductivity due to the percolation mechanism.

The heat transfer between colliding nanostructures may increase the thermal conductivity of the nanofluids. Particularly, a higher temperature corresponds to a more intense Brownian motion [38]. Thus, at 323 K, the thermal conductivity of nanofluids is more apparent than at the other evaluating temperatures. Moreover, liquid layering at the nanostructures/fluid can also contribute to the improvement of thermal conductivity behavior [74,75,76].

In the hybrid nanofluids, it was observed that at room temperature, the effect of coupling h-BN and MoS_2_ was similar as a single component reinforcement, either h-BN or MoS_2_. Moreover, as evaluating temperature increased, the value of thermal conductivity was higher than the single component nanofluids [77,78]. h-BN contributed to the increase of the thermal conductivity as well as MoS_2_, promoting the dispersion of the nanostructures due to their lubricant properties [38,79].

## 4. Conclusions

Thermal transport phenomena in nanofluids was influenced by physical and chemical characteristics of the base fluid, as well as interactions with the reinforcement nanostructures. Environmentally-friendly nanofluids based on single h-BN, single MoS_2_, and hybrid h-BN/MoS_2_ nanostructures were developed at various filler fractions, and temperature-dependence evaluations were performed for thermal conductivity. In general, all nanofluids showed a temperature-dependence in thermal conductivity performance, indicating the role of the interaction of nanostructures among synthetic and natural esters. The addition and homogeneous dispersion of these 2D nanomaterials within conventional esters showed significant positive results on the effective thermal conductivity performance. Thermal conductivity improved in the range of 21–23% for single reinforcement of h-BN or MoS_2_ on natural esters and 23–27% for synthetic esters at 323 K, respectively. Furthermore, for the hybrid h-BN/MoS_2_ nanofluids, an improvement of 30–32% was observed for natural esters and 32% for synthetic esters at 323 K, respectively. Hence, the hybrid h-BN/MoS_2_ nanostructures dispersed in conventional esters induced a high thermal conductivity, suggesting that the hybrid h-BN/MoS_2_ have highly desirable multifunctional features for advanced materials applications.

This research showed the potential of an interesting synergistic effect resulting from the contribution of two 2D nanostructures for synthetic and natural esters. Increased environmental awareness is the main driving force for the development of novel technologies, such as the use of biodegradable fluids and lubricants in environmentally-sensitive areas, which have great potential to succeed in industrial applications.

## Figures and Tables

**Table 1:** Material properties [56,57,58,59].

General Properties	Materials				
	Units	Standard	Envirotemp ^®^ FR3 ^TM^	Midel 7131	VG-100
Density @ 25 °C	g/cm^3^	ASTM D924	0.92	0.95	0.92
Kinematic viscosity @ 25 °C	mm^2^/s	ASTM D445	-	53	-
Kinematic viscosity @ 40 °C	mm^2^/s	ASTM D445	33	29.5	31
Kinematic Viscosity @ 100 °C	mm^2^/s	ASTM D445	8	5.3	6
Thermal conductivity @ 25 °C	(W m^−1^ K^−1^)	ASTM D7896	0.167	0.147	-
Thermal conductivity @ 50 °C	(W m^−1^ K^−1^)	ASTM D7896	-	0.145	-
Pour point	(°C)	ASTM D97	−18 to −23	−56	−12 to −15

**Table 2:** Nanostructures characteristics at 298 K.

General Properties	h-BN	MoS_2_	Units
Purity	98	99.98	%
Density	2.29	5.06	gr/cm^3^
Particle size		5.0–10.0	μm
Thermal conductivity	300–500 [60]	38–131 [61,62]	W/m K
